# The Role of the Advanced Practice Nurse in Survivorship Care Planning

**DOI:** 10.6004/jadpro.2015.6.1.7

**Published:** 2015-01-01

**Authors:** Virginia Sun1, Jill M. Olausson2, Rebecca Fujinami1, Carrie Chong3, Rachel Dunham4, Tami Tittlefitz5, Kelly Greer1, Marcia Grant1

**Affiliations:** 1Division of Nursing Research & Education, Department of Population Sciences; 2Department of Diabetes, Endocrinology and Metabolism; 3Division of Surgical Oncology, Department of Surgery; 4Tobacco Exposure and Lung Cancer Screening Program; 5Division of Thoracic Surgery, Department of Surgery, City of Hope, Duarte, California

Comprehensive survivorship care planning (SCP) is an essential component of quality cancer care. Since the 2006 seminal publication of the Institute of Medicine’s report on cancer survivorship, it has been unclear which models of cancer survivorship care translate to improved patient and system outcomes. An emerging model of survivorship care that has great potential for improving outcomes involves advanced practitioners, including advanced practice nurses (APNs) and physician assistants (PAs), as key providers of care for cancer survivors following completion of primary treatment.

The purpose of this article is to describe the role of the APN in SCP through a brief review of the literature and two case studies focused on survivors with colorectal and non–small cell lung cancer. More than 40% of people born in the United States today will be diagnosed with cancer at some point in their lives (McCabe, Partridge, Grunfeld, & Hudson, 2013).

An estimated 14.5 million Americans with a history of cancer were alive in 2014; this number is expected to increase to nearly 19 million by 2024 (DeSantis et al., 2014). In 2006, the Institute of Medicine (IOM) released a seminal report, "From Cancer Patient to Cancer Survivor: Lost in Transition," that identified cancer survivorship as a distinct phase of cancer care that has been neglected in important areas such as clinical guidelines, research, and advocacy (Hewitt, Greenfield, & Stovall, 2006).

There is considerable debate among stakeholders as to who should be responsible for developing and providing a personalized plan for posttreatment care and what should be considered when developing that plan. It has become clear that the model used to care for a cancer survivor population is dependent upon the setting in which the care is provided, the cancer diagnosis and/or treatment, and the resources available to the practice or institution, as well as a host of other factors (McCabe & Jacobs, 2012).

A widely utilized model is the APN-driven survivorship care program (Gates, Seymour, & Krishnasamy, 2012; McCabe & Jacobs, 2012). The holistic nursing focus in conjunction with clinical knowledge and skills makes the APN well suited to coordinate high-quality, patient-centered, transitional care. The purpose of this article is to illustrate, through two case study exemplars, the role of the APN as the key provider of comprehensive cancer survivorship care.

## SURVIVORSHIP CARE PLANNING AND ADVANCED PRACTICE NURSING

The 2006 IOM report recognized four essential components of patient-centered survivorship care: (1) prevention of recurrent and new cancers and other late effects; (2) surveillance for cancer spread, recurrence, or second cancers and assessment of medical and psychosocial late effects; (3) interventions for consequences of cancer and its treatment; and (4) coordination between specialists and primary care providers (PCPs) to ensure that all of the survivor’s health needs are met (Hewitt, Greenfield, & Stovall, 2006).

Additionally, in 2011, the LIVESTRONG Foundation facilitated a consensus of key stakeholders (survivors, physicians, and nurse practitioners) in cancer survivorship care that delineated 20 essential elements of cancer survivorship programs (see Table 1). These were divided into three tiers: what survivorship providers must include (tier 1), *should* include (tier 2), and *should strive* to include (tier 3) in their approach to care (Rechis, Beckjord, Arvey, Reynolds, & McGoldrick, 2011).

**Table 1:**
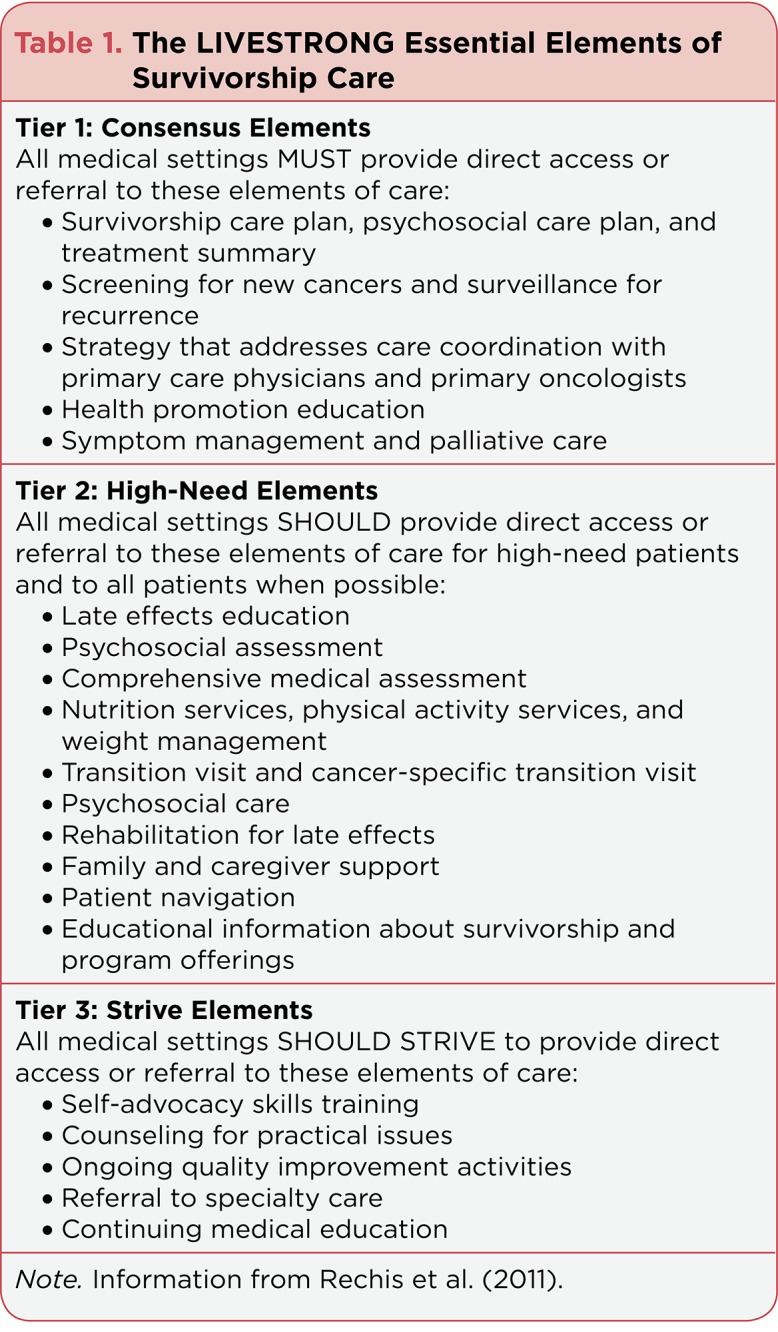
The LIVESTRONG Essential Elements of Survivorship Care

Both the IOM report and the LIVESTRONG consensus statements recommend that cancer patients completing primary treatment be provided with a comprehensive summary of their treatment together with a follow-up care plan, as part of comprehensive survivorship care planning (SCP; Hewitt, Greenfield, & Stovall, 2006). Though evidence supporting the efficacy of SCP is still being accumulated through research, the American College of Surgeons Commission on Cancer has included accreditation requirements related to the inclusion of treatment summaries and care plans into its Cancer Program Standards for 2015 (American College of Surgeons, 2012). These standards, which include the mandate that survivorship care plans be prepared by oncology providers and provided to survivors at the completion of treatment, are widely expected to lead to increasing uptake of the integration of quality survivorship care into standard cancer care (Stricker & O’Brien, 2014).

Many descriptive studies have assessed the perspectives of stakeholders involved in SCP. A comprehensive review by Salz and colleagues (2012a) summarized these findings and noted that survivors expressed needs across many topics in survivorship care. Informational needs identified by survivors include a written list of treatments received and their cancer diagnosis, recommendations for follow-up care, and clarification of who is responsible for this care. Survivors also wanted information on the physical and psychological long-term and late effects of treatment, prevention of recurrences, second cancers, other cancer-related health problems, as well as general health promotion strategies. Additionally, survivors reported that survivorship care plans empowered them to guide their own care and to ensure that they receive recommended screenings (Stricker et al., 2011).

In studies describing PCP perceptions of survivorship care, it was noted that PCPs report a general discomfort when caring for cancer survivors and felt SCP that included a concise diagnosis and treatment summary, guidelines, and recommendations for survivorship care, treatment complications, and patient-specific recommendations for managing late effects would be of benefit (Salz et al., 2012a). Oncologists also reported that they would be uncomfortable providing survivorship care and that SCP would be helpful (Forsythe et al., 2012; Klabunde et al., 2013). Oncologists believed SCP could be useful to survivors by reducing anxiety and increasing communication between health-care providers. However, implementation challenges from the oncologist’s perspective included having available personnel and resources to complete each individual plan and managing reimbursement issues (Jacobs et al., 2009; Mayer, Gerstel, Leak, & Smith, 2012; McCabe, Partridge, Grunfeld, & Hudson, 2013).

In order to provide cancer survivors with the recommended and required posttreatment care, community and comprehensive cancer centers across the nation have been individually developing SCP models of care. Individual survivor characteristics and the wide variety of local and contextual health-care environments in which survivorship care is delivered precludes a "one-size-fits-all" approach to care (Howell et al., 2012; McCabe & Jacobs, 2012). Some studies have attempted to test the efficacy of the various models: nurse-led, PCP-led, specialist- or oncologist-led, or shared care (Howell et al., 2012). When testing the difference between nurse-led follow-up care and oncology-led care, no significant differences were found between quality of life (QOL) and disease recurrence. Moreover, survivors reported improvement in satisfaction and emotional functioning in one of the nurse-led SCP studies (Moore et al., 2002).

Advanced practitioners are the key providers of care for many adult survivorship clinical models being instituted nationally, sometimes practicing in an independent role or, in other situations, working as part of a multidisciplinary team. The case studies that follow provide an exemplar for the specific role that APNs can play in providing quality, evidence-based cancer survivorship care.

## CASE STUDY 1

Mr. K is 64-year-old Chinese male diagnosed with T3N1 rectal adenocarcinoma at an outside facility in January 2012. He has no family history of cancer. Past medical history includes arrhythmia and hypercholesterolemia. He is married, and he and his wife live locally. Mr. K was treated with neoadjuvant chemoradiation and then underwent a laparoscopic lower anterior resection with a diverting loop ileostomy. Adjuvant chemotherapy included eight cycles of FOLFOX (leucovorin, fluorouracil, and oxaliplatin) before he had a surgical takedown of his ileostomy.

At the SCP visit, Mr. K began the conversation immediately with concerns about his bowel incontinence and frequency. The APN discussed the management of these complications and provided Mr. K with a referral to physical rehabilitation and nutritional services. The APN used this opportunity to discuss other long-term effects of Mr. K’s cancer treatments listed in his survivorship care plan. She reviewed these effects and discussed recommendations and resources for their management. After Mr. K’s concerns were met, the APN then explained the overarching purpose of the survivorship care plan, beginning with the treatment summary. Mr. K stated that the survivorship care plan would be helpful to bring to his primary care appointment coming up next month.

Next, the APN reviewed Mr. K’s anticipated follow-up schedule for the first year (computed tomography [CT] scan, colonoscopy, lab work, medical and surgical oncology visits) as well as the expectations for longer-term follow-up. Mr. K was counseled on health promotion (physical activity, healthy diet) and other cancer screenings. The session then concluded with an in-depth review of local and national cancer survivorship resources that Mr. K might find helpful.

## CASE STUDY 2

Mr. B is a 50-year-old, married, self-employed mechanic who was diagnosed with stage IIA squamous cell non–small cell lung cancer 14 months ago at his local community hospital. He is an ex-smoker (50 pack-year history) with a history of substance abuse, chronic obstructive pulmonary disease, hypertension, and mild gastroesophogeal reflux disease. Mr. B was initially treated with definitive chemoradiation; surgery was not pursued due to poor pulmonary function. A posttreatment CT demonstrated increase in size of his right lower lobe mass. He was therefore referred to a cancer center located 2 hours from his home for further evaluation. At the cancer center, Mr. B completed preoperative pulmonary rehabilitation to optimize his pulmonary function and then proceeded to have an open bilobectomy.

The APN met with Mr. B at 1 month postsurgery to discuss SCP. During the visit, Mr. B identified problems with dyspnea, fatigue, anxiety, and mood. He said that he had been feeling down because he could not perform his usual job functions and he was having great difficulty being socially and sexually active with his wife. The APN screened Mr. B for depression and reassured him that what he was feeling was commonly experienced by cancer survivors. She let him know that he could receive help from mental health services. She also gave him a referral to pulmonary rehabilitation to reinforce techniques to alleviate dyspnea and fatigue. Mr. B expressed interest in receiving both services, but he was concerned that his insurance would not cover either one. The APN let Mr. B know that supportive care services at the cancer center would be contacting him to help with insurance and financial concerns and to recommend resources for services within his area. After concluding her conversation with Mr. B, she faxed referrals to supportive services.

Within 2 months of his last postoperative visit, Mr. B experienced cough, dyspnea, and fever that resulted in him going to a local emergency room (ER) for assessment and treatment. Following treatment for an upper respiratory tract infection, a CT scan of the chest was recommended. Mr. B checked his care plan and noted that he had a CT scheduled in 1 month. He asked the local ER provider to call his surgical oncologist for his recommendation regarding doing the CT scan early. The CT was done at the local hospital and the results were sent to the cancer center.

## DISCUSSION

The cases of Mr. K and Mr. B illustrate the multiple functions that APNs provide during SCP, including the provision of care plans, management of late and long-term effects of treatment, counseling on general health promotion strategies, and the coordination and communication of care among members of the health-care team (see Table 2). In the first case, the APN developed a plan of care based on the survivor’s treatment history and potential sequelae from these treatments. At the beginning of the review visit, the APN assessed that Mr. K had pressing concerns regarding bowel incontinence. The APN was able to use adult learning principles (Bastable, 2008) to address these concerns first before focusing on the additional information presented as part of comprehensive SCP. The APN was able to coordinate care through communication with Mr. K’s oncology team and change his short-term follow-up schedule so it would be more convenient for him. In the second case, care was also tailored to patient-specific needs, not only meeting the plan for follow-up and surveillance but also taking into consideration Mr. B’s psychosocial and financial concerns.

**Table 2:**
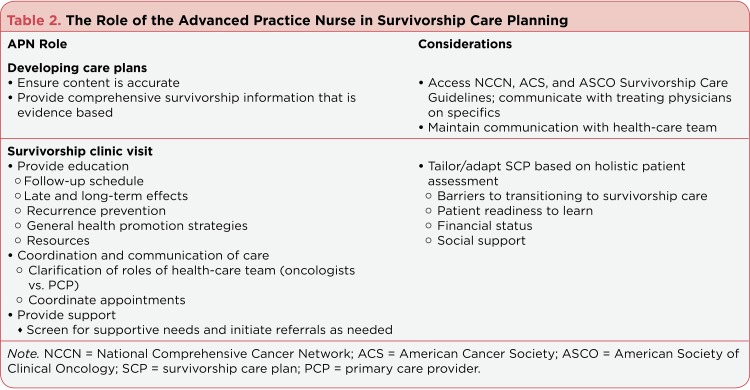
The Role of the Advanced Practice Nurse in Survivorship Care Planning

The APN-driven SCP model facilitated high-quality cancer care in both of these examples. Both cases showed that the "teachable moment," when the APN engaged the patient in his SCP, did more than just provide education, communication, and care coordination (Demark-Wahnefried, Aziz, Rowland, & Pinto, 2005; Ganz, 2005; McBride & Ostroff, 2003). It also encouraged each patient to take a more active role in his survivorship care, similar to results found in other studies (Ganz, 2005; McBride & Ostroff, 2003; McCorkle et al., 2011; Salz et al., 2012b). When Mr. K communicated his concerns about his bowel incontinence to the APN, he was taking an active role in tailoring his care.

Patient engagement was demonstrated in the second case study as well. Because Mr. B knew from his care plan that he had a CT scheduled at the cancer center in the next month, he was able to contact his oncologists and have his CT scan sent from his local hospital to the cancer center, thus avoiding duplication in services.

Both Mr. K and Mr. B expressed overwhelming satisfaction with their SCP and felt that it involved them in the plan of care. Self-advocacy skills training has been identified by the LIVESTRONG Essential Elements of Survivorship Care as an element that providers should strive to incorporate in SCP (Rechis et al., 2011). Self-advocacy is an important component of self-management in chronic illness (Jonikas et al., 2013), including the chronicity of cancer survivorship (McCorkle et al., 2011), and may prove to be an integral and significant benefit of SCP.

## IMPLICATIONS FOR ADVANCED PRACTICE

Advanced practitioners, including APNs, are increasingly being recognized as key providers of SCP either independently or as part of a multidisciplinary team. In order to facilitate high-quality SCP, APNs will need to combine their foundational APN knowledge with oncology-specific evidence-based guidelines. A comprehensive assessment and screening of specific survivorship care needs is an important component of SCP. The APN must consider a survivor’s physical and psychosocial characteristics to tailor their care. This assessment, together with oncology-specific knowledge and skills and the actual and potential effects of treatments, can further personalize the plan of care.

The Figure provides an adapted version of the City of Hope quality-of-life model that illustrates the potential physical, psychological, social, and spiritual sequelae of colorectal and non–small cell lung cancer and treatment (Ferrell, Dow, Leigh, Ly, & Gulasekaram, 1995). The APN must also have knowledge of cancer-specific surveillance and healthy living recommendations. Table 3 provides links to survivorship care guidelines from the National Comprehensive Cancer Network (2013) and the American Cancer Society (2013) guidelines for healthy living.

**Figure 1:**
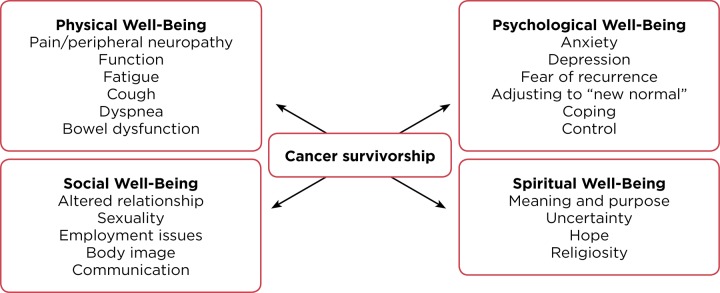
"Figure. City of Hope quality-of-life model applied to colorectal and non–small cell lung cancer survivorship. Information from Ferrell et al. (1995).

**Table 3:**
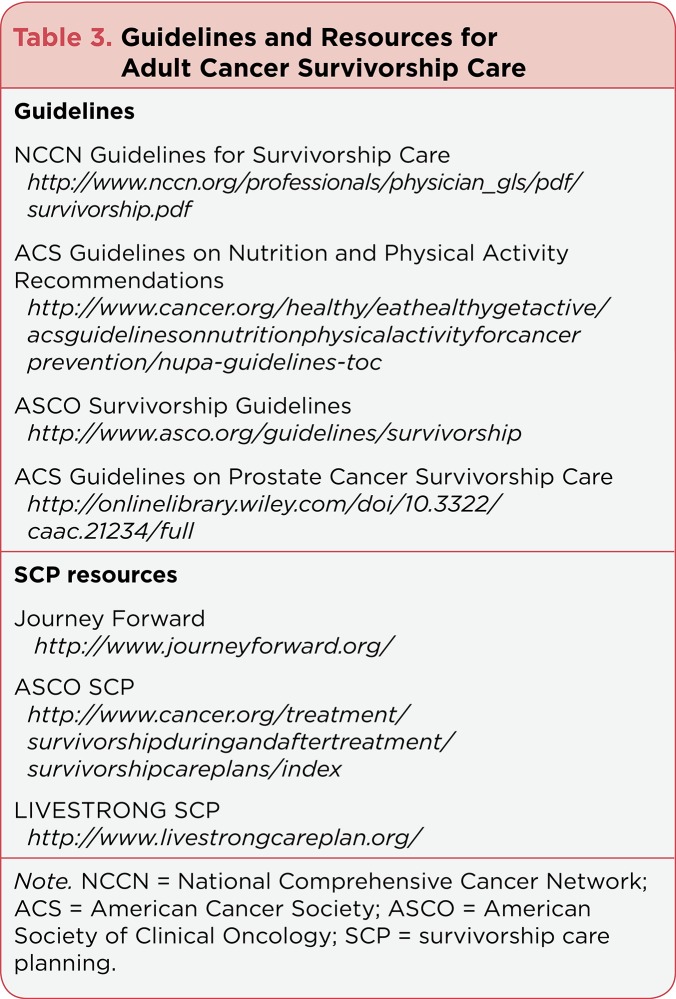
Guidelines and Resources for Adult Cancer Survivorship Care

In addition to general and disease-specific knowledge, APNs should be aware of their state’s scope of practice for APNs as well as practice patterns at their facility. Because of the real and expected shortage of oncology practitioners to meet the growing survivor population (Erikson, Salsberg, Forte, Bruinooge, & Goldstein, 2007), it will become increasingly important for APNs to understand and advocate for their role in SCP.

## CONCLUSIONS

Individual survivor, disease, and treatment characteristics are just as important as the health-care context when planning the transition from acute oncology care to chronic survivorship care. The case studies presented in this article illustrate some ways in which APNs are able to plan care for survivors by using general and specific advanced practice knowledge. While the role of the APN in SCP is under development, APNs (and other advanced practitioners) have the potential to provide SCP that is in alignment with national recommendations and guidelines for quality cancer survivorship care.
